# Patatin-like phospholipase domain-containing 3 (PNPLA3) variants rs 738408 and rs 738409 single nucleotide polymorphism as predictor of metabolic associated fatty liver disease and its progression

**DOI:** 10.12669/pjms.41.5.11532

**Published:** 2025-05

**Authors:** Benash Altaf, Shireen Jawed, Wan Syaheedah Wan Ghazali, Ahmad Aizat Abdul Aziz, Rana Muhammad Tahir Salam, Abid Rasheed, Mahaneem Mohamed

**Affiliations:** 1 Benash Altaf, (MBBS, MPhil) Aziz Fatimah Medical and Dental College, Faisalabad, Pakistan; 2 Shireen Jawed, (MBBS, MPhil) Frontier Medical and Dental College, Abbottabad, Pakistan; 3 Wan Syaheedah Wan Ghazali, (MD,PhD) Department of Physiology, School of Medical Sciences, Universiti Sains Malaysia; 4 Ahmad Aizat Abdul Aziz, Human Genomic Center, School of Medical Sciences, Universiti Sains Malaysia; 5 Rana Muhammad Tahir Salam, (MBBS) Sonologist, Aziz Fatimah Hospital, Faisalabad, Pakistan; 6 Abid Rasheed, Government College University, Faisalabad, Pakistan; 7 Mahaneem Mohamed, (MD,PhD) Department of Physiology, School of Medical Sciences, Universiti Sains Malaysia

**Keywords:** Adiponutrin, Genetic variants, Metabolic associated fatty liver disease, PNPLA3, rs738408, rs 738409

## Abstract

**Objective:**

To identify the PNPLA3 genetic variants as the potential predictors for metabolic associated fatty liver disease (MAFLD) and its progression among Pakistani population.

**Method:**

A cross sectional study comprising of 158 participants. They were included in this study with non-probability purposive sampling technique from Radiology Department of Aziz Fatimah Teaching Hospital and Aziz Fatimah Medical and Dental College, Pakistan. The duration of study was December 5, 2021 to April 4, 2024. Blood samples (10mL) were drawn for liver enzymes, gene analysis and ultimately PCR products were sent for Sanger sequencing. Data was analyzed using SPSS version 26 with p-Values ≤0.05 considered significant.

**Results:**

From 158 studied participants, 43.7% were male, and 56.3% were female. Of total population 51.3% were MAFLD positive while 48.7% were control subjects. Present results showed that CT and TT genotypes of rs738408 were frequently found in MAFLD group (p=0.039 and 0.060, respectively) and significant predictor for developing MAFLD (OR=2.484, p=0.019 and OR=5.167, p<0.001, respectively). Concerning progression of the disease CT genotype showed that subject with this genotype progress to moderate grade of fatty liver disease (OR=2.832, p=0.013). Whereas Odd ratio for TT genotype was significant for severe grade fatty liver diseases (OR=15.50, p=0.007). GG genotype of rs738409 was frequently found in MAFLD group (p=0.032). Odds ratio for GG genotype of rs738409 was significant for developing MAFLD (OR=4.565, p=0.025) and responsible to progress to moderate grade of fatty liver disease (OR= 5.083, p=0.022).

**Conclusion:**

CT and TT genotypes of 738408 and GG genotype of 738409 are associated with MAFLD and are at more risk for disease progression.

## INTRODUCTION

Metabolic associated fatty liver disease (MAFLD) is a new concept, replacing non- alcoholic fatty liver disease (NAFLD), introduced as it is associated with a range of various conditions (obesity, hyperlipidemia, and diabetes mellitus that are caused by accumulation of fat in liver).^1^ All the risk factors of metabolic syndrome including obesity, deranged lipid profile, hypertension and diabetes can aggravate the disease process. Outcomes of the disease are significantly poor including hepatic cirrhosis, failure and carcinoma and making it the leading reason for liver transplant.^2^ As, this concept has been finalized in late 2020 to replace the previously known term NAFLD to MAFLD.^3^ Hence, this new term MAFLD has been used throughout this study.

MAFLD has surfaced the global health issue as its prevalence is hiking continuously which is expected to even increase dramatically by 2030, additionally calling attention to stark increase in prevalent cases of decompensated cirrhosis, incident cases of liver cancer, and the need for liver transplantation.^4^ Previous literature report showed that the prevalence of MAFLD was 36.8% in Mediterranean region, 20%–40% in Europe, 9%–30% in Japan, 16%–32% in Indian urban, 5%– 24% in China while 5% in Singapore.^5^ However according to current data the prevalence of MAFLD in Pakistan is 28.9%.^6^

Fatty liver in metabolic associated disorders thereby comprising MAFLD, is diagnosed using either an imaging or a liver biopsy in individuals with at least 5% of hepatocytes infiltrated with steatosis who consume little or no alcohol and without any secondary causes of hepatic steatosis, such as other metabolic liver diseases (e.g., Wilson’s disease, congenital or acquired lipodystrophy) or drugs (e.g., amiodarone, steroids, tamoxifen, valproic acid, etc.).^7^

PNPLA3 gene, encodes a protein called adiponutrin. It is located at long arm of chromosome 22 q13.31 of hepatocytes and adipocytes.^8^ The PNPLA3 has the enzyme activity that is found in hepatocytes and adipocytes, and believed to play a crucial role for hepatic lipid remodeling.^9^ The mutation of this gene i.e. PNPLA3 I 148M was the first major gene associated with fatty liver eventually lead to more severe liver diseases such as cirrhosis and hepatocellular carcinoma.^10^ Moreover, the PNPLA3 rs 738408 and rs 738409 variants affects lipid droplet–Golgi dynamics, causing significant morphological changes in the Golgi apparatus, including increased lipid droplet–Golgi contact sites. These changes are observed in primary human patient hepatocytes expressing the I148M variant.^10^ The expression of PNPLA3-I148M also induces proteomic and transcriptomic changes that resemble all stages of liver disease, further highlighting its role in disease progression.^10^ It is the most replicated modifier of MAFLD pathogenesis in different ethnicities.^11^

The wild type PNPLA3 has function of triglyceride hydrolase and acetylCoA-independent transacylase activity, and loss of its function consequently leads to an accumulation of triglycerides droplets and retinyl esters within hepatocytes and cause endoplasmic reticulm (ER) stress and damage to liver cells eventually leading to fatty liver.^12^ Studies have shown that loss of function of PNPLA3 leads to increased hepatic steatosis and elevated serum alanine aminotransferase (ALT) and aspartate aminotransferase (AST) levels. Patients with MAFLD who carry the PNPLA3 rs738409 C/G mutation, a genetic polymorphism, characterized by the substitution of isoleucine to methionine at position 148 (I148M), are more likely to develop steatohepatitis and fibrosis. This nonsynonymous variant or missense mutation is the main genetic risk factor for disease severity and progression.^13^ Previous studies consistently showed a strong association between I148M and hepatocellular triglyceride accumulation, increased fibrosis and severity of steatohepatitis with genotype rs 738409.^14^

However, data concerning rs 738409 and rs 738408 is lacking in Pakistan particularly targeting the association of these SNPs with progression of MAFLD. On the other hand, only one Iranian data concerning rs 738408 and its association with a condition of MAFLD is available, but this data also lacks concerning progression of MAFLD and its association with these SNPs. So, current study aimed to identify the PNPLA3 genetic variants as the potential predictors for MAFLD and its progression among Pakistani population.

## METHODS

This cross-sectional study was a joint project of Universiti Sains Malaysia and Aziz Fatimah Teaching Hospital and Aziz Fatimah Medical and Dental College, Pakistan from December 5, 2021 to April 4, 2024. Using the non-probability purposive sampling technique, sample 158 subjects including male and female of age ranged from 30 - 80 years were enrolled from Aziz Fatimah Teaching Hospital and Aziz Fatimah Medical and Dental College, Faisalabad, Pakistan. The ethical approval (USM/JEPem/2109063, Ref. No.: IEC/122-21, dated: June 2, 2022) was taken from the respective hospital.

Sample size was calculated by PS software. The assumption of Type-I error probability (p) was 5% (0.05) and the power of the study was 80% (0.8) for all parameters. The mean difference for serum AST, ALT, TG between two groups (δ) were 6.0, 9.0and 45, respectively, while within group standard deviation (σ) were 11.7, 19.11, and 97.94, respectively, based on previous studies.^15^ The ratio of control to the experimental group (m) was standardized at one. The largest calculated sample size was 75 subjects. Considering the 15% drop-out rate, the sample size for each group was 86 subjects. However, the final number of subjects for each group was 77 after excluding the dropped-out subjects for control group and 81 for MAFLD group, comprising a total population size of 158 participants. Subjects with fatty liver, diagnosed on ultrasound were enrolled that were presented with one of the three following criteria i.e. overweight/ obesity, presence of Type-2 diabetes mellitus, or evidence of metabolic dysfunction including interrelated biochemical abnormalities like central obesity, dyslipidemia and hypertension as subjects in MAFLD group were included. Metabolic dysfunction was defined as the presence of two or more of these conditions: (1) Waist circumference >95th percentile for age and gender, (2) Blood pressure >95th for age, gender, and height. (3) Triglycerides >150 mg/dL, (4) HDL < 40 mg/dl, (5) C-reactive protein (CRP) levels>2 mg/L.^3^,^16^ All non-diabetic subjects determined by fasting blood glucose level ≤ 126 mg/dL, age-matched normotensive and healthy non-diabetic normal weight individuals without fatty liver as subjects in control group.

Diagnosis of fatty liver was made upon finding on ultrasound with high echogenic texture indicating fatty liver by expert sonologist and graded as mild, moderate and severe depending upon the degree of hepatic echogenicity with renal cortex, portal vein and diaphragm, respectively.^17^ Subject positive for viral serology (HBsAg, anti HCV antibodies), metabolic dysregulation i.e. endocrine, thyroid, calcium, or prediabetics with fasting blood glucose 100mg-125 mg/dL were excluded from study. Moreover, subjects with history of taking any hakeem, homeopathic or hepatotoxic drugs like paracetamol, tetracyclines, steroids, anti-tuberculous drug i.e. pyrazinamide, anti-inflammatory drugs, 3-hydroxy-3-methylglutaryl-coenzyme A (HMG-CoA) reductase inhibitors, valproic acid, carbamazepine, chemotherapy and heavy alcoholics (consumption of > 20 mg/day alcohol) were also excluded from the study.

Demographic and anthropometric data (height, weight, waist circumference and body mass index (BMI)), were collected using standard protocol. History of hypertension and diabetes was also taken and confirmed on fasting blood sugar level. Serum biochemistry including ALT, AST, triglycerides and total cholesterol were analyzed with spectrophotometry. For each subject, 10 mL blood was taken under aseptic measure, of which five mL was used for biochemical analysis after centrifuging it while another five mL of blood was stored at -20 C till analysis for DNA analysis.

For DNA analysis, DNA was extracted from whole blood by using DNA mid kit (Thermo Scientific GeneJET Genomic DNA Purification Kit K0722) using manufacturers protocol with gel tubes. Quality of DNA was checked by 1% agarose gel electrophoresis in 1*TAE buffer and confirmed by Nanodrops (1.0-2.0). Then, conventional PCR was done for amplification and 333bp fragment of exon were identified using Thermo Scientific DreamTaq PCR Master Mix (2X) K 1081 on gel as followed with primers sequences F: 5-TGGGCCTGAAGTCCGAGGGT-3′and R: 5′- CCGACACCAGTGCCCTGCAG-3.^18^ Purified PCR product of 333bp along with forward primer were sent for sanger sequencing (Macrogen, Korea). Sanger sequence analysis was carried out using Bio Edit software.

### Statistical analysis:

All collected data was first entered in MS excel and shifted to SPSS version 26 for analysis. Categorical data is presented as frequency and percentages while numerical data is presented in mean ± SD. Normality of the data was checked by Kolmogorov-Smirnov for quantitative variables. It was observed that the data for all quantitative variables were deviating from normality for both groups with p-Values <0.05 except for cholesterol and weight which were normally distributed with p-Value ≥ 0.05. So we applied non-parametric, Mann-Whitney U test for comparing the skewed variables among case and control. Chi-square used to compare the proportions. Whereas parametric independent t-test was used for normally distributed variables. Odds ratio were calculated using binominal as well multinomial logistic regression analysis for estimation of the risk for MAFLD and progression of disease, respectively. A p-Value of ≤0.05 was considered as statistically significant.

## RESULTS

We recruited 158 subjects, out of which 89(56.3%) and 69(43.7%) were female. Out of total subjects 77(48.7%) were control and 81(51.3%) were having fatty liver disease. From the recruited subjects in the present study, 77(48.7%) subjects were normal, 16(10.1%) were of mild fatty liver, 55(34.8%) were of moderate fatty liver and 10(6.3%) were of severe grade fatty liver. Age, anthropometric and biochemical data of MAFLD and control groups are shown in Table-I. In MAFLD group, BMI value was found statistically significantly higher as compared to control group (p>0.000). However, no significant differences were found for waist circumference, hip circumference as well as waist and hip ratio between the two groups. Comparison of normally distributed variables including cholesterol and weight were analyzed by Independent t-test. No significant difference was noted concerning total serum cholesterol and weight among the studied groups (Table-I). To fulfill MAFLD criteria, 45 (55.6%) were hypertensive while 40(49.4%) were diabetic and 62 (76.5%) were obese subjects.

**Table-I:** Distribution of anthropometric and biochemical variables among MAFLD and control groups by status.

Parameters	MAFLD (n=81)		Control (n=77)		p-Value
	Mean SD	Median(IQR)	Mean SD	Median(IQR)	
Age (year)	48.92±3.90	49.00(11.82)	50.27±11.98	50.00(19.50)	0.40^#^
Weight (kg)	74.5432±11.39	78.00 (17.00)	74.77±14.50	74.00(17.00)	0.90^##^
BMI kg/m2	28.56±6.69	27.46(7.40)	24.23±5.40	23.7(4.37)	0.000^#^
Waist cm	38.86±3.78	38.00(2.50)	38.64±3.35	38.00(2.00)	0.97^#^
Hip cm	41.38±3.90	41(2.50)	41.28±3.83	41(2.00)	0.91^#^
Waist /hip	0.93±0.56	0.95(0.05)	0.93±0.38	0.95(0.04)	0.55^#^
Height (m)	6.14±5.76	5.40(0.50)	5.32 ±0.52	5.40(0.65)	0.68^#^
ALT (U/L)	38.33±21.975	30(17.00)	28.32±13.329	28(19.50)	0.20^#^
AST (U/L)	55.44±38.90	41(34.00)	36.89±19.78	38(26.00)	0.15^#^
CH (mg/dl)	168.03±46.12576	160(50.00)	156.91±35.99813	169(49.50)	0.52^##^
TG (mg/dl)	227.62±95.88	181(94.50)	180.37±93.25	183(150.00)	0.96^#^
ALT/AST	0.78±0.206	0.76(0.24)	0.81±0.19	0.760(0.17)	0.52^#^

# Mann-Whitney U test;

## Independent T- test;. **BMI:** body mass index; **WHR:** waist-hip ratio; **TG:** triglycerides; **ALT:** alanine transferase; **AST:** aspartate transaminase; **CH:** cholesterol. p-Value ≤ 0.05 was taken as statistically significant.

As shown Table-II, the present study results revealed that the CC wild type genotype of rs 738408 was frequently found in normal healthy subjects in contrast to MAFLD (80.5% vs 59.3%, respectively). Heterozygous CT genotype was most frequently found in MAFLD subjects as compared to control group (30.9% vs 16.9%, respectively), and similarly homozygous variant TT was also frequently found among the MAFLD subjects in contrast to control healthy subjects (9.9% vs. 2.6%, respectively). Statistically significant differences were observed in percentages of CC and CT genotypes of rs738408 among MAFLD and control groups of study with p-Values= 0.004 and 0.039, respectively. However, no significant difference was noted for TT genotype (p-Value= 0.060). C allele was most frequently found in control group as compared to MAFLD (0.89% vs 0.75%, respectively), whereas T allele was frequently present in MAFLD group than control (0.25% vs 0.11%, respectively). These findings were found to be statistically significant p-Value with P value =0.001 (Table-II).

**Table-II:** The genotype and allele frequency of PNPLA3 (rs 738408 & rs 738409) in MAFLD patients and Healthy controls.

PNPLA3 SNP		Control Group (n=77)		MAFLD Group (n=81)		p-Value
SNPs738408		Frequency	%	Frequency	%	
CC (wild Type)		62	80.5	48	59.3	0.004*
CT		13	16.9	25	30.9	0.039*
TT		2	2.6	8	9.9	0.060
*Alleles*		*count*	%	*count*	%	.001*
Alleles 738408	C	137	0.89	121	0.75	
	T	17	0.11	41	0.25	
*SNP 738409*		*Frequency*	*%*	*count*	*%*	
CC (wild Type)		61	79.2	49	60.5	.010*
CG		13	16.9	21	25.9	0.167
GG		3	3.9	11	13.6	0.032*
	*Control Group=77*		*MAFLD Group =81*			
Allele 738409		count	%	count	%	
	C	135	0.88	119	0.73	.001*
	G	19	0.12	43	0.27	

MAFLD: Metabolic associated fatty liver disease; SNP: single nucleotide polymorphism. Statistical analysis was done using Chi-Square test. p-Value ≤ 0.05 was taken as significant.

Concerning genotype rs738409 frequencies and percentages, the present study showed that the CC wild type allele was frequently found in normal healthy subjects as compared to MAFLD subjects (79.2% vs 60.5%, respectively). Heterozygous CG genotype was most frequently found in MAFLD subjects as compared to Control group (25.9% vs 16.9%, respectively), and similarly homozygous variant GG was also frequently found among the MAFLD subjects in contrast to healthy control subjects (13.6% vs 3.9%, respectively) (Table-II). Statistically significant difference was observed in percentages of these of CC and GG genotypes of rs 738409 among MAFLD and control groups of study with p-Values 0.010 and 0.032, respectively. However, no significant difference was noted for CG genotype (p-Value= 0.167). C allele was most frequently found in control group as compared to MAFLD group (0.88% vs 0.73%, respectively), whereas G allele was frequently present in MAFLD group than control (0.27% vs 0.12%, respectively). These finding were found to be statistically significant with p-Value 0.001 (Table-II).

Binary logistic regression was used to predict rs 738408 and rs 738409 for estimation of risk for MAFLD. Odds ratio was calculated using CC wild type as a reference value. A significant odd ratio of 2.484 at 95% CI for CT genotype of 738408 was found as a risk for having MAFLD, (p-Value=0.019). At the same time, a significant odd ratio of 5.167 was also found for TT genotype (p-Value<0.001) (Table-III). Odds ratio for evaluating the association for rs 738409 with MAFLD was calculated using CC wild type as a reference value. Odds ratio of 2.011 at 95% CI was noticed for CG genotype of 738409 as a risk for having MAFLD, (p-Value 0.081). However, OR for GG genotype was statistically significant with p-Value 0.025 (Table-III).

**Table-III:** PNPLA3 variants 738408 and 738409 SNPs as risk for developing MAFLD n=158

PNPLA3 gene	Genotype	Control group (n=77)		MAFLD Group (n=81)		OR (95% CI)	p-Value
		Frequency	%	Frequency	%		
SNPs rs 738408	CC	62	80.5	48	59.3	1 (Reference)	
	CT	13	16.9	25	30.9	2.484 (1.1514- 503588)	0.019*
	TT	2	2.6	8	9.9	5.167(1.049-25.455)	<0.001*
SNPs rs 738409	CC	61	79.2	49	60.5	1 (Reference)	
	CG	13	16.9	21	25.9	2.011 (0.915- 4.419)	0.081
	GG	3	3.9	11	13.6	4.565(1.026-17.273)	0.025*

MAFLD: Metabolic associated fatty liver disease; SNP: single nucleotide polymorphism; OD: odds ratio. p-Value ≤ 0.05 was taken as statistically significant using binary logistic regression.

Genetic risk for progression of various stages of fatty liver with rs 738408was analyzed using CC genotype (wild type) as reference value. A significant odds ratio of 2.832 for CT genotype of rs738408 was found as a risk for developing moderate grade of fatty liver disease (p-Value=0.013). Meanwhile, a significant odds ratio of 15.50 for TT genotype of rs738408 was found as a risk for developing severe grade of fatty liver disease (p-Value =0.007) (Table-IV).

**Table-IV:** Genetic risk for progression of various stages of fatty liver with rs 738408.

PNPLA3 variant			Odds ratio	95% Confidence Interval for Exp(B)		p-Value
			Lower Bound	Upper Bound
SNP 738408	CT	Mild FL	2.385	.698	8.148	.166
		Moderate FL	2.832	1.242	6.458	.013*
		Severe FL	.795	.088	7.172	.838
	TT	Mild FL	3.100	.257	37.452	.373
		Moderate FL	3.875	.673	22.303	.129
		Severe FL	15.500	2.149	111.782	.007*
SNP 738409	CG	Mild	2.346	.686	8.020	.174
		Moderate	2.200	.933	5.183	.071
		Severe	.670	.076	5.925	.719
	GG	Mild	2.033	.192	21.532	.556
		Moderate	5.083	1.261	20.494	.022*
		Severe	5.810	.824	40.943	.077

The reference category is CC. p-Value ≤ 0.05 was taken as significant using multinominal logistic regression. FL: Fatty Liver

Genetic risk for progression of various stages of fatty liver with rs 738409 using CC (wild type) genotype as reference value, showed a significant odds ratio of 5.083 for GG genotype having moderate grade of fatty liver disease (p-Value=0.022). However, no significant odds ratio for CG genotype was found for progression to various stages of fatty liver disease (Table-IV). Results of chi-square concerning the association of SNP rs 738408 and SNP rs 738409 with biochemical parameters are shown in Table-V, indicating that these parameters are not significantly associated with various genotype rs738408 and rs 738409.

**Table-V:** Association of biochemical variables with SNP rs738408 and SNP rs 738409 (n=158).

Biochemical variables	Status of biochemical variables	Genotype of rs 738408			p-Value
		CC (wild type) n (%) 110(69.6)	CT heterozygous variant n (%) 38 (24)	TT hemozygous variant n (%) 10(6.3)	
CH	normal	95(86.4%)	30(78.9%)	8(80%)	0.52
	abnormal	15(13.6%)	8 ( 21.1%)	2(20%)	
TG	normal	37(33.6%)	12 (31.6%)	3(30%)	0.94
	abnormal	73(66.4%)	26 (68.4%)	7(70%)	
ALT	normal	32(29.1%)	15(39.5%)	4(40%)	0.43
	abnormal	78(70.9%)	23(60.5%)	6(60%)	
AST	normal	62(56.4%)	28(73.7%	5(50%	0.13
	abnormal	48(43.6%)	10(26.3%	5(50%
*Biochemical variables*	*Status of biochemical variables*	*Genotype of rs 738409*			
		*CC (wild type) n (%) 110(69.6)*	*CG hetrozygous variant n (%) 34(21.5)*	*GG homozygous variant n (%) 14(8.8)*	*p-Value*
CH	normal	95(86.4)	28(82.4)	10(71.4)	0.33
	abnormal	15(13.6)	6(17.6)	4(28.6)	
TG	normal	37(33.6)	11(32.4)	4(28.6)	0.92
	abnormal	73(66.4)	23(67.6)	10(71.4)	
ALT	normal	32(29.1)	12(35.3)	7(50)	0.26
	abnormal	78(70.9)	22(64.7)	7(50)	
AST	normal	61(55.5)	26(76.5)	8(57.1)	0.08
	abnormal	49(44.5)	8(23.5)	6(42.9)	

Chi-square test was applied. p-Value ≤ 0.05 was taken as significant. CH: cholesterol; TG: triglycerides;

## DISCUSSION

This study was conducted to insight the predictors of development of MAFLD and progression of fatty liver into its severe stages. Previous studies have highlighted the importance of variant rs 738408 and rs 738409 for MAFLD^18^ but to date still no study in Pakistan has been done to predict rs 738408 as a risk factor for MAFLD. Furthermore, associations of 738408 and 738409 genotypes for progression of fatty liver disease is still lacking to date. Though International literature documented the risk of PNPLA3 rs738409 as the predictors for MAFLD but the present study for the first time proved the role of PNPLA3 rs 738408 in MAFLD and its association with progression of fatty liver disease and documenting this relation in Pakistan.

Result of present study concerning demographic and liver enzymes for MAFLD and Control showed that MAFLD subjects had significantly higher BMI with value as compared to control group subjects. However, no significant differences between waist circumference, hip circumference and waist-hip ratio were observed. The present study is in-line with the Asian study conducted in Iranian population, which also found higher BMI and other demographics variables in MAFLD subjects.^18^ Aforementioned study also found higher levels of ALT, AST, cholesterol, TG and other biochemical variables in MAFLD subjects which were also in line with the present study conducted in Pakistan revealing higher levels of biochemical parameter but values were not found to be statistically significant.^18^

While heterozygous CT allele was most frequently found in MAFLD subjects as compared to control group (30.9% vs 16.9%, respectively), and similarly homozygous variant TT was also frequently found among the MAFLD subjects in contrast to control healthy subjects (9.9% vs. 2.6%). A statistically significant difference was observed in percentages of these genotypes of rs738408 among MAFLD and control groups of study (*p*-value= 0.01). Previous study conducted in Asia is in line with the present results showing greater number of genotype CC in control group as compared to MAFLD group. However, TT and CT genotypes were higher in percentage in MAFLD group as compared to control.^18^ According to aforementioned literature, for rs 738408, TT and TC were associated with MAFLD (p=0.004)^18^ which are in line with our results indicating that subjects with rs 738408, CT and TT genotypes are at two times more risk for developing MAFLD as compared to control group (OR= 2.305, *p*-Value= 0.024).

Frequency and percentage of PNPLA3 variant 738409 in healthy control and MAFLD subjects reveals that the CC wild type genotype was frequently found in normal healthy subjects as compared to MAFLD subjects (79.2% vs 60.5%, respectively). Heterozygous CG genotype was most frequently found in MAFLD subjects as compared to control group (25.9% vs 16.9%, respectively), and similarly homozygous variant GG was also frequently found among the MAFLD subjects in contrast to healthy control subjects (13.6% vs 3.9%, respectively). A statistically significant difference was observed in percentages of these alleles of rs 738409 among MAFLD and control groups of study. While study conducted in Iran and Turkey showed that CC was less frequently present in MAFLD subjects as compared to control subjects, while GG and CG were present in higher percentages in MAFLD subjects as compared to control subjects.^18^,^19^

SNP 738408 as a risk factor in MAFLD and control groups was analyzed by binary logistic regression suggesting that the subjects with CT genotype will have 0.194 times more risk for developing MAFLD (*p*-Value=0.004). Till date, only one study available in literature showing that subjects rs 738408 CT and TT genotypes are at more risk of developing MAFLD as compared to CC wild type. Concerning the present results for rs 738409 as a risk in MAFLD showed that CG genotype has significant odds ratio and have two times risk for developing MAFLD as compared to control group. However, the present results did not find significant odds ratio for GG genotype as a risk for MAFLD.

Iranian study found both genotypes rs738409 GG or GC as a risk for MAFLD.^18^ Similar to these results, Turkish study also found that subjects with both homozygouse GG variant and heterozygous CG variant are associated with risk of developing MAFLD [2.462 (1.253–4.834) p=0.01, 2.432 (1.198–4.935) p=0.05].^19^ A Pakistani study also reported 3% of GG genotype patients was associated with hepatic steatosis.^20^ In line with the findings of the present study, several studies also showed that CT heterozygous genotype of SNP 738408 and CG genotype of SNP 738409 were significantly associated with MAFLD condition^21^, showing that subjects with these genotypes have risk to develop fatty liver.^21^ A study conducted in Karachi, Pakistan also reported hepatic steatosis in subjects with CG and GG genotypes of rs 738409.^22^

Genetic risk of 738408 for progression of various stages of fatty liver revealed significant odds ratio for CT was 2.832 showing that subjects having CT wild type have 2.8 times more risk than CC wild genotype for developing moderate grade of fatty liver disease (*p*-Value=0.013). For SNP 738408, the significant odds ratio for TT genotype was 15.50 showing that subjects having TT genotype have almost 15 times more risk than normal CC genotype for developing severe grade of fatty liver disease. Genome-wide association analyses in UK showed that CT is responsible for poor fatty liver outcomes.^23^ For genetic risk for progression of various stages of fatty liver, significant odds ratio for GG genotype was 5.083 showing that subjects having GG allele have 5 times more risk than normal CC genotype for developing moderate grade of fatty liver disease. However, study in UK also found risk of poor fatty liver outcome with rs 738409 for C/G.^23^

With compliance to the present results, Pennisi et. al., (2021) also reported 16%, 19%, and 32.8% risk for development of MAFLD with PNPLA3 CC/CG/GG genotype of rs 738409, respectively.^24^ However, meta-analysis conducted by Nader et. al., (2021) also showed that subjects with GG genotype were more at risk for MAFLD. In Pakistan, a study also found a significant odds ratio for GG genotype.^25^ Hence, the PNPLA3 gene (rs738409) plays a significant role in the progression of fatty liver disease.

Among the MAFLD subjects, association of biochemical parameters with SNP rs738408 and rs 738409 with Chi-square test analysis indicated that these parameters were not significantly associated with various genotypes of rs738409 in the present study. Similar finding was observed by the previous literature, reporting no significant association between rs 738409 with biochemical parameters.^18^ However, no literature is available to date for rs 738408 and its association with biochemical markers.

### Strength of study and clinical relevance:

PNPLA3 gene has been identified as a key genetic factor in the development and progression of fatty liver disease, influencing lipid accumulation and cellular changes that can lead to advanced liver conditions including cirrhosis and hepatocellular carcinoma. Hence, timely identification of these genotypes in subjects would target them as a risk population for this disease and would help them to seek timely precautions.

### Limitations:

Although the study is conducted in the largest private sector tertiary care hospital, it was considered as a single centered study in which the results cannot be generalized to the whole population. Hence, further researchers should be conducted on broader scale to identify which genotype is a risk factor for fatty liver disease and its progression in whole district and subsequently in whole region. Moreover, targeting these genes for therapeutic purpose and on pharmaceutical grounds can even open new horizons in the field of hepatic diseases.

## CONCLUSION

In Pakistani population, PNPLA3 variant rs 738408 with CT and TT genotype, and rs 738409 with GG genotype are associated with MAFLD and are at more risk for progression of this fatty liver disease towards its severity.

### Recommendation:

Moreover, it is suggested that implementing preventive strategies by spreading awareness campaigns, investing in research, and preparing better health care system, we can minimize the impact of this disease and improve the quality of life of millions individuals affected by this liver disease.

### Author’s contributions:

**BA:** Conceptualization, Data Collection, Data Analysis - Original Draft, Visualization, manuscript preparation, revised and approved the final version.

**SJ:** Data Collection, Data Analysis, revised and approved the final version

**WSWG:** Study design, Analysis, Data Analysis, Writing Review, Revised and approved the final version

**AAAA:** Data analysis, Writing - Review & Editing the manuscript, Revised and the final version

**RMTS**: Study design, data collection, Review & Editing Revised and approved the final version **AR:** Study design**,** Review & Editing, Revised and the final version

**MM:** Conceptualization, Supervision, Original Draft Preparation, Visualization, Editing, revised and the final version.

All authors are equally responsible for the integrity of the data.

## References

[1] Kaya E, Yilmaz Y (2021). Metabolic-associated Fatty Liver Disease (MAFLD):A Multi-systemic Disease Beyond the Liver. J Clin Transl Hepatol.

[2] Fassio E, Barreyro FJ, Pérez MS, Dávila D, Landeira G, Gualano G (2022). Hepatocellular carcinoma in patients with metabolic dysfunction-associated fatty liver disease:Can we stratify at-risk populations?. World J Hepatol.

[3] Eslam M, Newsome PN, Sarin SK, Anstee QM, Targher G, Romero-Gomez M (2020). A new definition of metabolic dysfunction-associated fatty liver disease:An international expert consensus statement. J Hepatol.

[4] Dong X, Li JM, Lu XL, Lin XY, Hong MZ, Weng SPJ (2024). Global burden of adult non-alcoholic fatty liver disease (NAFLD) and non-alcoholic steatohepatitis (NASH) has been steadily increasing over the past decades and is expected to persist in the future. Transl Gastroenterol Hepatol.

[5] Ramesh T, Appandraj S (2020). Prevalence of Non-Alcoholic Fatty Liver Disease and Associated Factors among Type 2 Diabetics of Kanchipuram:A Hospital Based Cross-Sectional Study. IAIM.

[6] Hassan F, Farman M, Khan KA, Awais M, Akhtar S (2024). Prevalence of nonalcoholic fatty liver disease in Pakistan:a systematic review and meta-analysis. Nature.

[7] Mazoa DF, Maltac FM, Stefanoa JT, Salles APM, S GGM, Nastri ACS (2019). Validation of PNPLA3 polymorphisms as risk factor for NAFLD and liver fibrosis in an admixed population. Ann Hepatol.

[8] BasuRay S, Smagris E, Cohen JC, Hobbs H (2017). The PNPLA3 variant associated with fatty liver disease (I148M) accumulates on lipid droplets by evading ubiquitylation. Hepatology.

[9] Barbara M, Scott A AN (2018). New Insights Into Genetic Predisposition and Novel Therapeutic Targets for Nonalcoholic Fatty Liver Disease. Hepatobiliary Surg Nutr.

[10] Sherman DJ, Liu L, Mamrosh JL, Xie J, Ferbas J, Lomenick B (2017). The fatty liver disease–causing protein PNPLA3- I148M alters lipid droplet–Golgi dynamics. Proc Natl Acad Sci.

[11] Anstee QM, Day CP (2015). The Genetics of Nonalcoholic Fatty Liver Disease:Spotlight on PNPLA3 and TM6SF2. Semin Liver Dis.

[12] Heeren J, Scheja L (2021). Metabolic-associated fatty liver disease and lipoprotein metabolism. Mol Metab.

[13] Liu M, Park S (2024). The Role of PNPLA3 _rs738409 Gene Variant, Lifestyle Factors, and Bioactive Compounds in Nonalcoholic Fatty Liver Disease:A Population-Based and Molecular Approach towards Healthy Nutrition. Nutrients.

[14] Albhaisi S, Sanyal AJ (2021). Gene-Environmental Interactions as Metabolic Drivers of Nonalcoholic Steatohepatitis. Front Endocrinol.

[15] Mansour-Ghanaei R, Mansour-Ghanaei F, Naghipour M, Joukar F (2019). Biochemical markers and lipid profile in nonalcoholic fatty liver disease patients in the PERSIAN Guilan cohort study (PGCS), Iran. J Family Med Prim Care.

[16] Katsiki N, Perez-Martinez P, Anagnostis P, Mikhailidis DP, Karagiannis A (2018). Is Nonalcoholic Fatty Liver Disease Indeed the Hepatic Manifestation of Metabolic Syndrome?. Curr Vasc Pharmacol.

[17] Paul J (2020). Recent advances in non-invasive diagnosis and medical management of non-alcoholic fatty liver disease in adult. Egyptian Liver J.

[18] Najafi M, Rafiei A, Ghaemi A, Hosseini V (2022). Association between rs738408, rs738409 and rs139051polymorphisms in PNPLA3 gene and non-alcoholic fatty liver disease. Gene Reports.

[19] Akkiz H, Taskin E, Karaogullarindan U, Delik A, Kuran S, Kutlu O (2021). The influence of RS738409 I148M polymorphism of patatin-like phospholipase domain containing 3 gene on the susceptibility of non-alcoholic fatty liver disease. Med.

[20] Jalil I, Arshad M, Khan S, Dasti JI (2020). PNPLA3 and TM6SF2, but Not MBOAT7, Are Associated with Steatosis and HBV Viral Persistence in Pakistani Population. undishapur J Microbiol.

[21] Ali A, Amin MJ, Ahmed MU, Taj A, Aasim M, Tabrez E (2022). Frequency of non-alcoholic fatty liver disease (NAFLD) and its associated risk factors among Type-2 diabetics. Pak J Med Sci.

[22] Siyal M, Abbas Z, Saeed A, Qadeer MA (2024). Mesenteric panniculitis associated with non-alcoholic fatty liver disease:A case series. J Pak Med Assoc.

[23] Chen VL, Chen Y, Du X, Handelman SK, Speliotes EK (2020). Genetic variants that associate with cirrhosis have pleiotropic effects on human traits. Liver Int.

[24] Pennisi G, Pipitone RM, Cammà C, Di Marco V, Di Martino V, Spatola F (2021). PNPLA3 rs738409 C>G Variant Predicts Fibrosis Progression by Noninvasive Tools in Nonalcoholic Fatty Liver Disease. Clin Gastroenterol Hepatol.

[25] Nader S, Niloufar D, Mansouri K, Far HGM, Darvishi F, Mohammad M (2021). Association between PNPLA3 rs738409 polymorphism and nonalcoholic fatty liver disease:a systematic review and meta-analysis. Endocr Disord.

